# Cardiovascular Disease Prevalence in Non-Hispanic Black Individuals: U.S. Territories vs. Stroke Belt and Non-Stroke Belt States

**DOI:** 10.7759/cureus.72215

**Published:** 2024-10-23

**Authors:** Hana Shah, Namra Khan, Carolina Fernandez, Ligia Perez, Georgeta D Vaidean

**Affiliations:** 1 Herbert Wertheim College of Medicine, Florida International University, Miami, USA; 2 Institutional Transformation Assessment, Gardner Institute, Brevard, USA

**Keywords:** cardiovascular disease, geographical region, health disparities, non-hispanic black adults, non-stroke belt states, race, stroke belt states, u.s. territories

## Abstract

Introduction: Extensive research exists on the increased prevalence of cardiovascular disease (CVD) in the “Stroke Belt” states, compared to the remaining United States (U.S.). Social, environmental, and systemic factors and individual behaviors have been investigated, including Black race.  This study aims to assess whether residing in the U.S. territories, Stroke Belt, or non-Stroke Belt states is associated with differences in CVD prevalence among non-Hispanic Black adults.

Methods: We analyzed cross-sectional data from the 2021 Behavioral Risk Factor Surveillance System (BRFSS). All non-Hispanic Black adults (18+) with complete information on key variables, including demographics, CVD outcomes, and relevant risk factors, were included. The prevalence of CVD was defined as self-reported coronary artery disease, stroke, or myocardial infarction. Univariate and multivariable logistic regression analyses were used to calculate crude and adjusted odds ratios (ORs) with 95% confidence intervals (CIs).

Results: Of the 31,246 individuals included in our study, 87 (8.7%) residing in the U.S. territories reported experiencing a cardiovascular event, compared to 1487 (10.1%) in the Stroke Belt and 1872 (8.2%) in the non-Stroke Belt regions. Compared to non-Hispanic Black adults residing outside the Stroke Belt, those living in the Stroke Belt had 23% higher odds of reporting CVD (OR 1.23, 95% CI 1.10-1.44) after adjusting for age and gender. In the fully adjusted model, which accounted for additional factors such as comorbidities and socioeconomic status, the odds were slightly attenuated but remained elevated (OR 1.14, 95% CI 0.97-1.35). For Black adults living in the U.S. territories, the odds of reporting CVD were not significantly different from those outside the Stroke Belt in both the age- and gender-adjusted model (OR 1.07, 95% CI 0.66-1.73) and the fully adjusted model (OR 0.93, 95% CI 0.49-1.74). Independent of residence, individuals with high blood pressure (OR 2.85, 95% CI 2.05-3.96), diabetes (OR 1.45, 95% CI 1.20-1.75), and high cholesterol (OR 1.55, 95% CI 1.27-1.89) had significantly higher odds of CVD. In contrast, smoking and income were associated with prevalent CVD, while self-reported diet, physical activity, and education level were not.

Conclusion: Contrary to our expectation, we found that non-Hispanic Black adults residing in the U.S. territories had similar self-reported CVD with those living in the non-Stroke Belt regions.  Further research is needed to investigate the socio-behavioral factors influencing cultural and historical disparities among non-Hispanic Black individuals in the U.S. and its territories.

## Introduction

Cardiovascular disease (CVD) is the leading cause of morbidity and mortality globally. However, disparities in CVD serve as a reminder of the social injustices and inequities that persist [1]. Numerous studies found that people of color, particularly Black people, experience social disadvantages and barriers that increase CVD risk and lead to poor disease outcomes [1,2]. Black adults also have a higher burden of CVD risk factors, such as diabetes and hypertension, and are twice as likely to die from CVD, compared to White adults [2-4]. This racial disparity is further complicated by geographic differences within the United States (U.S.) [5].

A higher incidence of stroke and mortality has been reported among Black adults residing in the Southeastern region of the U.S., also known as the “Stroke Belt,” which includes Alabama, Arkansas, Georgia, Louisiana, Mississippi, North Carolina, South Carolina, Tennessee, and Virginia [5,6]. These states are included in the Stroke Belt due to their historically higher rates of stroke mortality and incidence compared to the rest of the country. While the higher concentration of Black individuals in this region partly contributes to the observed disparities, many studies suggest that health behaviors, socioeconomic factors, a history of slavery, and unequal access to healthcare and education are also significant drivers of the elevated risk of stroke in the Stroke Belt [5-8].

While numerous studies have highlighted the increased cardiovascular burden among Black adults, these studies are primarily confined to the Black American population residing in the 50 U.S. states and the District of Columbia (50 U.S./DC). Thus, little is known about the health of Black American people residing in the U.S. territories, which, for this study, include Puerto Rico (PR), Guam (GU), and the U.S. Virgin Islands (USVI) [9]. These territories were selected due to their distinct cultural, historical, and socioeconomic contexts, which differ from the U.S. mainland, including the Stroke Belt. By comparing CVD outcomes among Black adults residing in the U.S. territories, Stroke Belt, and non-Stroke Belt regions, we aim to better understand how these unique environments may influence cardiovascular health in this population.

This article was previously presented as a poster at the 2024 ACC Annual Scientific Session on April 6, 2024.

## Materials and methods

This was a cross-sectional study consisting of secondary data analysis from the Behavioral Risk Factor Surveillance System (BRFSS) database. The BRFSS is an annual, health-related telephone survey that collects state and local data about health-related risk behaviors, chronic health conditions, and the use of preventive services among residents of the 50 U.S./DC and U.S. territories. The mean combined response rate for landline and cell phone samples in 2021 was 44.6% (23.5% in GU, 49.7% in PR, 50.1% in USVI, and ranging from 23.5% to 60.5% in the U.S.) [10]. 

A total of 30,832 self-identified Black, non-Hispanic adults were included in this study, with 1128 (3.7%) individuals living in the U.S. territories, 11,289 (36.6%) living in the Stroke Belt areas, and 18,415 (59.7%) in the non-Stroke Belt areas. Geographic location was defined as follows: U.S. territories (PR, GU, and the USVI), “Stroke Belt” (Alabama, Arkansas, Georgia, Kentucky, Louisiana, Mississippi, North Carolina, South Carolina, Tennessee, and Virginia), and “non-Stroke Belt” (50 U.S./DC). Participants with missing information on race, state of residence, or cardiovascular events (5.9%) were excluded.

The primary outcome was the self-reported prevalence of CVD, defined as participants having ever been diagnosed by a healthcare provider with any of the following: coronary artery disease, stroke, angina pectoris, and/or myocardial infarction. We adjusted for 13 covariates across four domains. In the demographic information domain, we included age (categorized as 18-34, 35-49, 50-64, and 65+ years) and sex (male and female). The chronic conditions domain comprised self-reported diagnoses of high blood pressure (yes or no), diabetes (yes or no, without differentiation between type 1 and type 2), and total high cholesterol (yes or no). For lifestyle factors, we assessed BMI, categorized as normal (18.5-24.9 kg/m²), overweight (25.0-29.9 kg/m²), or obese (≥30 kg/m²); smoking status (current smoker, former smoker, never smoked); binge drinking (yes or no); and physical activity in the last 30 days (yes or no). Finally, in the socioeconomic factors domain, we considered recommended fruit and vegetable consumption (≥1 or <1 servings a day), education level (<high school, high school diploma, >high school), income level (<$50k, $50k-$100k, $100k to <$200k, ≥$200k), and healthcare access (yes or no).

Descriptive analyses were first conducted to summarize the distribution of these key characteristics among our study population, reporting means (standard deviations) and percentages as appropriate. Next, bivariate analysis using chi-square tests was performed to assess the association between residency (50 U.S./DC vs. U.S. territories) and the primary outcome of CVD prevalence. Subsequently, multivariable logistic regression analysis was employed to assess the relationship between residency and CVD, adjusting for potential confounders. This analysis followed a stepwise model-building approach: starting with a crude model, followed by minimally adjusting for age and sex, and then including the remaining 11 covariates. This method allows for a comprehensive evaluation of the influence of geographic residency on cardiovascular outcomes while controlling for confounding variables. Statistical significance was set at p < 0.05.

Lastly, we compared and reported CVD prevalence across residency groups (Stroke Belt vs. non-Stroke Belt vs. U.S. territories) in percentages to summarize the observed disparities in CVD outcomes among Black adults. All analyses were performed using the IBM SPSS Statistics for Windows, Version 28.0 (Released 2021; IBM Corp., Armonk, New York, USA).

## Results

Table 1 shows that age and sex were consistent across regions, but significant disparities were noted in several risk factors’ prevalence. High blood pressure was most common in the Stroke Belt areas (45.7%), while diabetes was highest in the U.S. territories (17.5%). Notably, the distribution of BMI showed substantial differences, with the highest levels observed in the Stroke Belt areas (47.2%). Behavioral factors exhibited regional differences, with 74.3% of individuals in the U.S. territories never having smoked, whereas binge drinking and fruit/vegetable consumption were not significant. Socioeconomic factors revealed that the U.S. territories had a higher percentage of individuals with less than high school education (27.9%) and lower incomes (86.1%), whereas the non-Stroke Belt areas had more individuals with education beyond high school.

**Table 1:** Baseline characteristics of non-Hispanic Black adults by region (2021 BRFSS) BRFSS: Behavioral Risk Factor Surveillance System.

	Geographic locations								
Characteristics	U.S. territories		Stoke Belt areas				Non-Stoke Belt areas		
	N	% weighted	N			% weighted	N	% weighted	p-value
Demographics									
Age groups (years)									0.375
18-34	207	31.3	1733			31.3	4133	30.5	
35-49	220	24.1	2308			25.9	4484	24.6	
50-64	366	25.2	3282			25.0	5248	26.1	
≥65	335	19.5	3966			17.8	4550	18.8	
Sex									0.195
Male	510	50.0	4426			46.8	8495	48.4	
Female	635	50.0	6992			53.2	10209	51.6	
Chronic diseases									
High blood pressure									<0.001
Yes	515	39.5	6603			45.7	8449	39.9	
No	626	60.5	4793			54.3	10204	60.1	
Diabetes									<0.001
Yes	201	17.5	2707			17.2	3152	14.3	
No	942	82.5	8692			82.8	15514	85.7	
Total high cholesterol									0.227
Yes	395	38.0	4199			33.7	5656	32.5	
No	620	62.0	5916			66.3	10292	67.5	
Body mass index (kg/m^2^)									<0.001
Normal weight	294	19.8	2302			22.7	4589	25	
Overweight	425	38.8	3651			30.2	6353	34.8	
Obese	426	41.4	5465			47.2	7762	40.2	
Behaviors									
Smoking status									<0.001
Current smoker	48	12.9	1707			17.8	2838	15.1	
Former smoker	136	12.7	2112			15.2	3368	17.1	
Never smoked	940	74.3	7211			67.0	11924	67.8	
Binge drinking									0.132
Yes	133	15.7	1072			12.6	2204	13.8	
No	945	84.3	9619			87.4	15248	86.2	
Exercise in the past 30 days									0.001
Yes	834	61.0	7699			71.1	13489	73.1	
No	311	39.0	3698			28.9	5179	26.9	
Fruit/vegetable consumption per day									0.057
≥1	891	77.8	8654			82.0	14213	83.4	
<1	178	22.2	1764			18.0	2756	16.6	
Socioeconomic factors									
Education level									<0.001
Less than high school education	164	27.9	1172			13.9	1354	11.4	
High school education	373	29.8	3528			32.8	5356	31.1	
Post-high school education	605	42.3	6695			53.2	11940	57.5	
Income level									<0.001
Less than $50,000	646	86.1	6017			60.7	8510	54.4	
$50,000 to less than $100,000	188	9.9	2259			25.2	4260	25.7	
$100,000 to less than $200,000	84	3.1	923			11.4	2281	15.7	
≥$200,000	30	0.9	176			2.7	631	4.1	
Healthcare access									0.032
Yes	957	85.6	10229			90.7	16709	91.8	
No	156	14.4	720			9.3	1097	8.2	

Table 2 examines confounding variables and their implications for CVD prevalence. Geographic location emerged as a significant factor, with the highest CVD prevalence in the Stroke Belt areas (10.1%), followed by the U.S. territories (8.7%), and the non-Stroke Belt areas (8.2%). Age was strongly associated with CVD prevalence, with older age groups 50-64 and ≥ 65 experiencing higher prevalence rates of 13.1% and 20.4%, respectively. However, CVD affects both genders similarly in these populations. Individuals with high blood pressure had a CVD prevalence of 17.1%, vs. 3% for those without. With the presence of diabetes, CVD prevalence rose from 6.7% to 21.5%, and similar trends were noted for hypercholesteremia. BMI was also noted as a critical contributor to CVD prevalence with higher rates observed among those classified as overweight (8.7%) and obese (10%). Behaviors such as smoking and lack of exercise also increased CVD events. Lower educational attainment and income were linked to higher CVD rates, while interestingly, individuals with access to healthcare experienced more CVD events (9.5%) compared to those without access (3.9%).

**Table 2:** Confounding variables and their relationship with cardiovascular disease prevalence

	Cardiovascular events				
Characteristics	Outcome present		Outcome absent		
	N	% weighted	N	% weighted	p-value
Geographic location					<0.001
U.S. territories	87	8.7	1058	91.3	
Stroke Belt areas	1487	10.1	9922	89.9	
Non-Stroke Belt areas	1872	8.2	16820	91.8	
Demographics					
Age groups (years)					<0.001
18-34	98	1.5	5974	98.5	
35-49	345	5.6	6662	94.4	
50-64	1150	13.1	7738	86.9	
≥65	1818	20.4	7028	79.6	
Sex					0.771
Male	1468	8.9	11945	91.1	
Female	1978	9.0	15855	91.0	
Chronic diseases					
High blood pressure					<0.001
Yes	2901	17.1	12658	82.9	
No	536	3.0	15078	97.0	
Diabetes					<0.001
Yes	1387	21.5	4670	78.5	
No	2050	6.7	23085	93.3	
Total high cholesterol					<0.001
Yes	2006	17.7	8240	82.3	
No	1193	6.1	15630	93.9	
Body mass index (kg/m^2^)					0.003
Normal weight	731	7.5	6447	92.5	
Overweight	1095	8.7	9328	91.3	
Obese	1620	10.0	12025	90.0	
Behaviors					
Smoking status					<0.001
Current smoker	684	13.0	3903	87.0	
Former smoker	1027	16.2	4585	83.8	
Never smoked	1624	6.1	18444	93.9	
Binge drinking					<0.001
Yes	240	6.0	3165	94.0	
No	3018	9.5	22784	90.5	
Exercise in the past 30 days					<0.001
No	1484	13.1	7694	86.9	
Yes	1955	7.4	20056	92.6	
Fruit/vegetable consumption per day					0.951
≥1	2615	9.0	21131	91.0	
<1	555	9.0	4139	91.0	
Socioeconomic factors					
Education level					<0.001
Less than high school education	560	17.6	2125	82.4	
High school education	1097	8.7	8151	91.3	
Post-high school education	1778	7.1	17456	92.9	
Income level					<0.001
Less than $50,000	2121	11.7	13045	88.3	
$50,000 to less than $100,000	449	5.6	6257	94.4	
$100,000 to less than $200,000	174	4.4	3114	95.6	
≥$200,000	34	3.6	803	96.4	
Healthcare access					<0.001
Yes	3180	9.5	24706	90.5	
No	110	3.9	1859	96.1	

Table 3 shows multivariable logistic regression models analyzing the association between geographic location and CVD prevalence. Initially, crude data indicate a significantly higher CVD prevalence in the Stroke Belt states compared to the non-Stroke Belt states (95% CI 1.10-1.44), but not in the U.S. territories. Model 1, adjusted for age and sex, shows a higher odds ratio (OR) for the Stroke Belt areas (OR 1.31, 95% CI 1.15-1.49) compared to the non-Stroke Belt areas, with no significant difference for the U.S. territories. As we adjust for more confounding factors in the subsequent models, the relationship between geographic location and cardiovascular events changes. In Model 2, which adjusted for chronic diseases, the OR for the Stroke Belt areas remains significantly higher (OR 1.21, 95% CI 1.05-1.39), while the OR for the U.S. territories remains nonsignificant. Model 3 adds behavioral factors to the previously adjusted models and shows consistent results. Model 4 further adjusts for socioeconomic factors, showing nonsignificant ORs for both the U.S. territories (OR 0.93, 95% CI 0.49-1.74) and Stroke Belt areas (OR 1.14, 95% CI 0.97-1.35). 

**Table 3:** Association between geographic location and prevalence of cardiovascular events ^a^Model 1 is adjusted for age and sex. ^b^Model 2 is adjusted for age, sex, hypertension, diabetes status, total high cholesterol, and BMI. ^c^Model 3 is adjusted for age, sex, hypertension, diabetes status, total high cholesterol, BMI, smoking status, binge drinking, exercise activity, and fruit/vegetable consumption. ^d^Model 4 is adjusted for age, sex, hypertension, diabetes status, total high cholesterol, BMI, smoking status, binge drinking, exercise activity, fruit/vegetable consumption, education level, income level, and healthcare access.

Geographical location	Crude	Model 1^a^ OR (95% CI)	Model 2^b^ OR (95% CI)	Model 3^c^ OR (95% CI)	Model 4^d^ OR (95% CI)
U.S. territories	1.07 (0.66-1.73)	1.05 (0.65-1.68)	1.00 (0.58-1.72)	1.05 (0.60-1.86)	0.93 (0.49-1.74)
Stroke Belt areas	1.23 (1.10-1.44)	1.31 (1.15-1.49)	1.21 (1.05-1.39)	1.22 (1.05-1.42)	1.14 (0.97-1.35)
Non-Stroke Belt areas	Ref.				

Table 4 summarizes the adjusted and unadjusted associations between selected covariates and CVD prevalence. Geographic location showed no significant differences in CVD prevalence between the U.S. territories and non-Stroke Belt areas in both unadjusted and adjusted models. However, residents in the Stroke Belt had higher CVD odds in the unadjusted analysis (OR 1.23, 95% CI 1.10-1.44), which became nonsignificant in the adjusted model. Increasing age was strongly associated with higher CVD prevalence, especially in those aged 65 and above (adjusted OR 4.50, 95% CI 2.86-7.10). Chronic diseases significantly increased CVD odds: high blood pressure (adjusted OR 2.85, 95% CI 2.05-3.961), diabetes (adjusted OR 1.45, 95% CI 1.20-1.75), and high cholesterol (adjusted OR 1.55, 95% CI 1.27-1.89). Behavioral factors showed that current smokers (OR 1.89, 95% CI 1.52-2.34) had higher CVD odds, while former smokers’ significance reduced (OR 1.50, 95% CI 1.15-1.93). Among the socioeconomic factors, individuals with less than a high school education and those with an income below $50,000 had significantly higher odds of CVD, even after adjustment. Lack of access to healthcare was significant in the unadjusted model, but after adjustment, this relationship became insignificant (OR 0.62, 95% CI 0.42-0.91). 

**Table 4:** Association between selected covariates and CVD prevalence CVD: cardiovascular disease.

Characteristics	Unadjusted		Adjusted	
	OR (95% CI)	p-value	OR (95% CI)	p-value
Geographic location				
U.S. territories	1.07 (0.66-1.73)	0.785	0.93 (0.49-1.74)	0.81
Stroke Belt areas	1.26 (1.10-1.44)	<0.001	1.14 (0.96-1.35)	0.13
Non-Stroke Belt areas	Ref		Ref	
Demographics				
Age groups (years)				
18-34	Ref		Ref	
35-49	3.95 (2.85-5.47)	<0.001	2.16 (1.40-3.35)	<0.001
50-64	9.96 (7.38-13.44)	<0.001	3.44 (2.26-5.24)	<0.001
65+	16.98 (12.55-22.96)	<0.001	4.50 (2.86-7.10)	0.008
Sex				
Male	0.98 (0.86-1.12)	0.771	1.05 (0.87-1.27)	0.635
Female	Ref		Ref	
Chronic diseases				
High blood pressure				
Yes	6.70 (5.38-8.34)	<0.001	2.85 (2.05-3.96)	<0.001
No	Ref		Ref	
Diabetes status				
Yes	3.83 (3.33-4.41)	<0.001	1.45 (1.20-1.75)	<0.001
No	Ref		Ref	
Total high cholesterol				
Yes	3.30 (2.86-3.81)	<0.001	1.55 (1.27-1.89)	<0.001
No	Ref		Ref	
Body mass index (kg/m^2^)				
Normal weight	Ref		Ref	
Overweight	1.17 (0.96-1.42)	<0.001	1.05 (0.80-1.38)	0.451
Obese	1.37 (1.17-1.60)	0.072	0.91 (0.72-1.15)	0.188
Behaviors				
Smoking status				
Current smoker	2.29 (1.92-2.72)	<0.001	1.89 (1.52-2.34)	<0.001
Former smoker	2.95 (2.48-3.52)	<0.001	1.50 (1.15-1.93)	0.002
Never smoked	Ref		Ref	
Binge drinking				
Yes	0.61 (0.49-0.76)	<0.001	0.84 (0.65-1.10)	0.209
No	Ref		Ref	
Exercise in the past 30 days				
Yes	Ref		Ref	
No	1.90 (1.66-2.17)	<0.001	1.14 (0.92-1.42)	0.225
Fruit/vegetable consumption per day				
≥1	Ref		Ref	
<1	1.00 (0.84-1.18)	0.951	0.91 (0.72-1.14)	0.392
Socioeconomic factors				
Education level				
Less than high school education	2.78 (2.26-3.43)	<0.001	1.37 (0.95-1.99)	0.095
High school education	1.25 (1.09-1.44)	0.002	0.95 (0.79-1.16)	0.627
Post-high school education	Ref		Ref	
Income level				
Less than $50,000	3.49 (1.90-6.43)	<0.001	2.63 (1.28-5.39)	0.008
$50,000 to less than $100,000	1.56 (0.83-2.92)	0.167	1.34 (0.65-2.77)	0.424
$100,000 to less than $200,00	1.22 (0.63-2.37)	0.557	1.19 (0.55-2.57)	0.657
≥$200,000	Ref		Ref	
Healthcare access				
Yes	Ref		Ref	
No	0.39 (0.29-0.53)	<0.001	0.62 (0.42-0.91)	0.013

## Discussion

The findings of our study highlight the significant regional disparities in CVD prevalence and associated risk factors among the Black adults residing in the U.S. and U.S. territories. Specifically, those residing in the Stroke Belt region initially exhibited a significantly higher CVD prevalence; however, after full adjustment for additional covariates such as socioeconomic factors, the disparities were reduced, highlighting the complex interplay of various risk factors. In our analysis, we employed a stepwise approach to model selection for multivariable logistic regression (Table 3). This method allowed us to evaluate the incremental impact of each set of covariates on the association between residency and CVD prevalence, ensuring that our final model adequately accounted for potential confounding variables. The inclusion of these covariates suggests that socioeconomic inequalities may play a more significant role than biological factors in driving the observed regional disparities. While Black American people are more prone to developing CVD risk factors, like diabetes and hypertension, the attenuation of these disparities highlights the impact of socioeconomic factors [5,6,11]. This emphasizes the complex interplay between geographic, behavioral, and sociodemographic factors and their role in shaping an individual’s cardiovascular health (Table 3). Additionally, our results reaffirm the impact of chronic diseases and health behaviors on CVD prevalence. Therefore, targeted strategies must be employed by healthcare professionals and policymakers to address the unique healthcare challenges faced by Black adults across the U.S. 

In examining demographic factors across different regions, it becomes evident that while certain aspects, such as age and sex, remain consistent, there are notable disparities in the prevalence of chronic diseases. Interestingly, studies have found that the cluster of cardiovascular comorbidities and stroke mortality in the Stroke Belt region may be partly rooted in historical factors, including the legacy of slavery and ongoing racial inequality. This has contributed to socioeconomic disadvantages, including limited access to healthcare and education, which continue to impact the health outcomes for Black populations in these areas [5]. Although BMI (47.2%) and high blood pressure (45.7%) were more prevalent in the Stroke Belt area, diabetes was unexpectedly higher (17.5%) in the U.S. territories. This discrepancy may reflect limitations in healthcare access, potentially leading to underdiagnosis or less effective management. Variations in lifestyle or unmeasured social determinants may also explain the discrepancy. To fully understand the relationship between BMI and diabetes, a more comprehensive exploration is needed, one in which unmeasured factors are evaluated. Significant differences in behaviors, like smoking and physical activity, were noted across the groups, emphasizing the role of regional factors on health. Those residing in the Stroke Belt and U.S. territories face unique geographical challenges that increase their CVD burden [8]. For instance, those in the Stroke Belt regions are surrounded by readily accessible fast-food options that contribute to the development of chronic health conditions like hypertension and obesity [2,12-16].

Hospitals in the U.S. territories had worse performance on core process measures and increased risk mortality and readmission rates compared to those in the U.S. However, it is important to note that the U.S. territories are diverse, with distinct cultural, economic, and healthcare challenges. These differences, such as variations in healthcare infrastructure and access to medical resources, likely contribute to the disparities in healthcare outcomes observed between the territories and both the Stroke Belt and other U.S. regions [15]. Our results also suggested that Black adults residing in the U.S. territories had the highest number of nonsmokers (74.3%), compared to the Stroke Belt (67%) and non-Stroke Belt (67.8%) areas. This may be indicative of better cardiovascular health profiles and healthier lifestyles seen in the territories [9]. On the other hand, residents in the U.S. territories experienced the highest poverty rates (86.1%) compared to the other U.S. regions. Together, these factors highlight the multifaceted nature of CVD prevalence and health disparities faced by the Black American population in the Stroke Belt and U.S. territories. 

While regional disparities exist, our analysis also highlighted the role of covariates on CVD prevalence. Comorbid conditions, like high blood pressure, diabetes, and high cholesterol, correlated with the increase in CVD prevalence, shedding light on the importance of effective chronic disease management. Our results indicated that CVD was more prevalent among people with less than a high school education or income below $50,000 [9,11,13]. This suggests that job requirements and economic factors in the region may deter some from pursuing a higher education [17]. Finally, Black American people with access to healthcare experienced higher CVD prevalence (9.5%) compared to those without access, suggesting excess use of healthcare services rather than effective preventive care. The findings underscore the need for public health interventions that proactively address risk factors, through screenings and early detection, to prevent diseases [18-21].

Our study findings need to be interpreted with several inherent limitations. Utilizing self-reported data from the BRFSS may introduce recall and nonresponse biases, which have the potential to underestimate associations. Additionally, the study’s cross-sectional design inherently limits causal inferences and understanding of the potential for temporal trends in CVD prevalence. Migration patterns, specifically of those moving to and from the studied region, could also influence CVD prevalence. For instance, those with higher socioeconomic status or better access to healthcare may have the opportunity to move out of the U.S. territories or Stroke Belt areas, underestimating the true burden of CVD in those areas. Since the BRFSS survey only provides a snapshot of health data at a single point in time, we were unable to track migration patterns. While our study provides valuable insights into regional disparities, further studies are warranted to analyze subtle distinctions in the U.S. territories, the impact of migration, and the influence of culture and socioeconomic factors on cardiovascular events. Specifically, longitudinal studies could help track health trends over time and evaluate the effectiveness of targeted interventions.

## Conclusions

Our study reveals notable disparities in the prevalence of CVD among Black individuals in the U.S. and its territories. We found that the non-Hispanic Black adults residing in the U.S. territories had similar self-reported CVD prevalence as those living in the non-Stroke Belt regions. However, the limitations of our study, such as reliance on self-reported data and the cross-sectional design, may affect the robustness of these conclusions and limit causal inferences. To achieve equitable cardiovascular health outcomes for all populations, it is crucial to address the various factors contributing to disparities in CVD. This includes not only biological risk factors but also socioeconomic determinants and historical contexts that perpetuate health inequalities. Public health programs should be employed to promote information on the management of chronic diseases, preventative healthcare, and socioeconomic assistance, particularly in the Stroke Belt regions and the U.S. territories. Given the complexity of these disparities, future research should focus on longitudinal studies that can track health trends over time and assess the impact of socioeconomic factors on CVD outcomes. Additionally, investigating the role of cultural determinants in shaping health behaviors will be essential for developing targeted interventions. By examining these intricate connections, we can provide insights to mitigate health inequalities and enhance the welfare of Black adults in diverse, understudied geographical areas. Ultimately, our findings reinforce the need for a multifaceted approach to public health that encompasses education, resource allocation, and community engagement.
